# Loss of anti‐spike antibodies following mRNA vaccination for COVID‐19 among patients with multiple myeloma

**DOI:** 10.1002/cnr2.1803

**Published:** 2023-03-09

**Authors:** Samuel D. Stampfer, Sean Bujarski, Marissa‐Skye Goldwater, Scott Jew, Bernard Regidor, Haiming Chen, Ning Xu, Mingjie Li, Eddie Fung, Regina Swift, Bethany Beatty, Shahrooz Eshaghian, James R. Berenson

**Affiliations:** ^1^ Division of Infectious Diseases, Department of Medicine Emory University School of Medicine Atlanta Georgia USA; ^2^ Institute for Myeloma and Bone Cancer Research West Hollywood California USA; ^3^ Berenson Cancer Center West Hollywood California USA; ^4^ Cedars Sinai Medical Center, Division of Hematology and Oncology Los Angeles California USA; ^5^ ONCOtherapeutics West Hollywood California USA

**Keywords:** antibodies, COVID‐19, half‐life, multiple myeloma, SARS‐CoV‐2, vaccination

## Abstract

**Background:**

Multiple myeloma (MM) patients have variable responses to mRNA vaccination to COVID‐19. Little is known regarding their vaccine‐induced antibody levels over time.

**Methods:**

We monitored spike IgG antibody levels over 24 weeks among a subset of 18 MM patients who showed a full response after two mRNA vaccinations.

**Results:**

MM patients had a more rapid decline in antibody levels as compared to eight healthy controls, with power law half‐lives of 72 days (vs. 107 days) and exponential half‐lives of 37 days (vs. 51 days). The patients with longer SARS‐CoV‐2 antibody half‐lives were more likely to have undetectable monoclonal protein than those with shorter half‐lives, suggesting better disease control may correlate with longer duration of vaccine‐induced antibodies. Regardless, by 16 weeks post‐second dose of mRNA vaccination, the majority of patients had antibody levels below 250 binding arbitrary units per milliliter, which would be unlikely to contribute to preventing COVID‐19.

**Conclusions:**

Thus, even MM patients who respond adequately to vaccination are likely to require more frequent booster doses than the general population.

## 1 INTRODUCTION

Vaccination for COVID‐19 reduces risk of severe disease and hospitalization.^1^ Unfortunately, immunocompromised individuals are less likely to develop a protective response to vaccination while simultaneously experiencing more complications from COVID‐19.^2^, ^3^, ^4^ One such group is patients with multiple myeloma (MM), a plasma cell dyscrasia resulting in excess production of monoclonal antibodies. These patients show impaired humoral immunity with reduced levels of uninvolved immunoglobulins; treatments for MM—typically geared toward suppressing malignant plasma cells—have off‐target effects that may further reduce B‐cell function and uninvolved antibody production. As a result, MM patients are at increased risk for infection.^5^ In our recently published study, only 45% of patients fully responded to mRNA vaccination for COVID‐19, which was considered a level above the 6th percentile of healthy controls.^3^ Thus, most patients remain susceptible to breakthrough infections despite vaccination. Unfortunately, widespread vaccination has failed to halt transmission,^6^ presenting an ongoing SARS‐CoV‐2 exposure risk for this patient population.

Quantitative anti‐spike antibody levels can be used to monitor vaccination responses with widespread use despite still‐yet‐undefined cutoffs for protection. Moreover, vaccine‐induced protection reduces with time,^1^ resulting in increased susceptibility to infection even among individuals who initially developed a fully protective antibody response.^7^ In this study, we monitored serial anti‐spike IgG levels to compare antibody decay in MM patients versus healthy controls.

## 2 METHODS

### 2.1 Antibody response to vaccination

Anti‐spike IgG was measured by semiquantitative ELISA.^3^ Following mRNA COVID‐19 vaccination, patients' serum antibodies were measured at serial intervals after their second dose: 2–3 weeks, 8–9 weeks, 16–17 weeks, and 23–24 weeks.

### 2.2 Participant selection

Participants included MM patients receiving care at a single oncology clinic specializing in the care of MM patients, as well as healthy controls that were typically spouses, friends or other family members of patients. To be able to accurately evaluate half‐lives of vaccine‐induced antibodies, only individuals who responded adequately to vaccination were included: those whose anti‐spike IgG levels increased at least 10‐fold from prevaccination to 2–3 weeks following their second dose (D2W2) with minimum levels of 250 BAU/mL (binding arbitrary units per milliliter calibrated to the WHO 20/136 international standard). Individuals were excluded if they were not evaluable at all of the time points or if they elected to obtain a booster vaccination prior to the end of the study. If individuals had evidence of SARS‐CoV‐2 exposure following their first vaccination, as defined by having an increase in anti‐spike antibody levels not attributable to vaccination, they were also not included in the study. Based on these criteria, 18 MM patients aged 61–84 (9 female, 9 male) and 8 controls aged 53–75 (3 male, 5 female) were evaluable. Individual participant information, including treatment regimen, is included in Table S1.

### 2.3 Half‐life calculations and statistical tests

Weeks 2 through 24 half‐lives were calculated using both exponential decay and power law methods.^8^ Comparisons on half‐lives between groups were analyzed using unpaired *t* tests due to normally distributed data. Comparisons between antibody levels were done by log‐transforming the antibody data (which has a lognormal distribution) and then comparing it with unpaired *t* tests. *Z* tests were used to compare the number of subjects in each response group (<50, 50–250, and >250 BAU/mL). An analysis of covariance (ANCOVA) test was done to assess whether half‐life differences between groups were still significant in spite of the controls being slightly younger. Tests were deemed statistically significant if *p* < .05. Analysis was done using R version 4.1.2 and GraphPad Prism 9.

## 3 RESULTS

**FIGURE 1:**
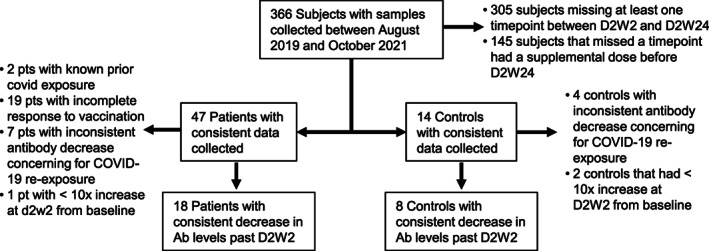
Participant flow diagram. Multiple myeloma (MM) patients and healthy controls were recruited at a single center specializing in plasma cell dyscrasias. Patients and controls were selected for half‐life analysis if they met all blood draw timepoints, developed an adequate response to vaccination (>250 BAU/mL), and had no evidence of re‐exposure to SARS‐CoV‐2 during the study (as evidenced by continuous decline in antibody levels at each subsequent analysis after D2W2).

MM patients and healthy controls were recruited from a single clinic specializing in treatment of this B‐cell malignancy. Initially, 48 patients and 11 age‐matched controls were recruited with planned follow‐up for 24 weeks. However, many patients and some controls independently elected to receive booster vaccinations prior to 24 weeks and were excluded from the analysis. Others had evidence of SARS‐CoV‐2 re‐exposure during the follow‐up period and were also excluded from half‐life calculations to avoid this confounder (Figure 1 **)**. This limited the study to 18 MM patients and 8 controls who were evaluable for their antibody decay following vaccination at all of the study time points.

**FIGURE 2:**
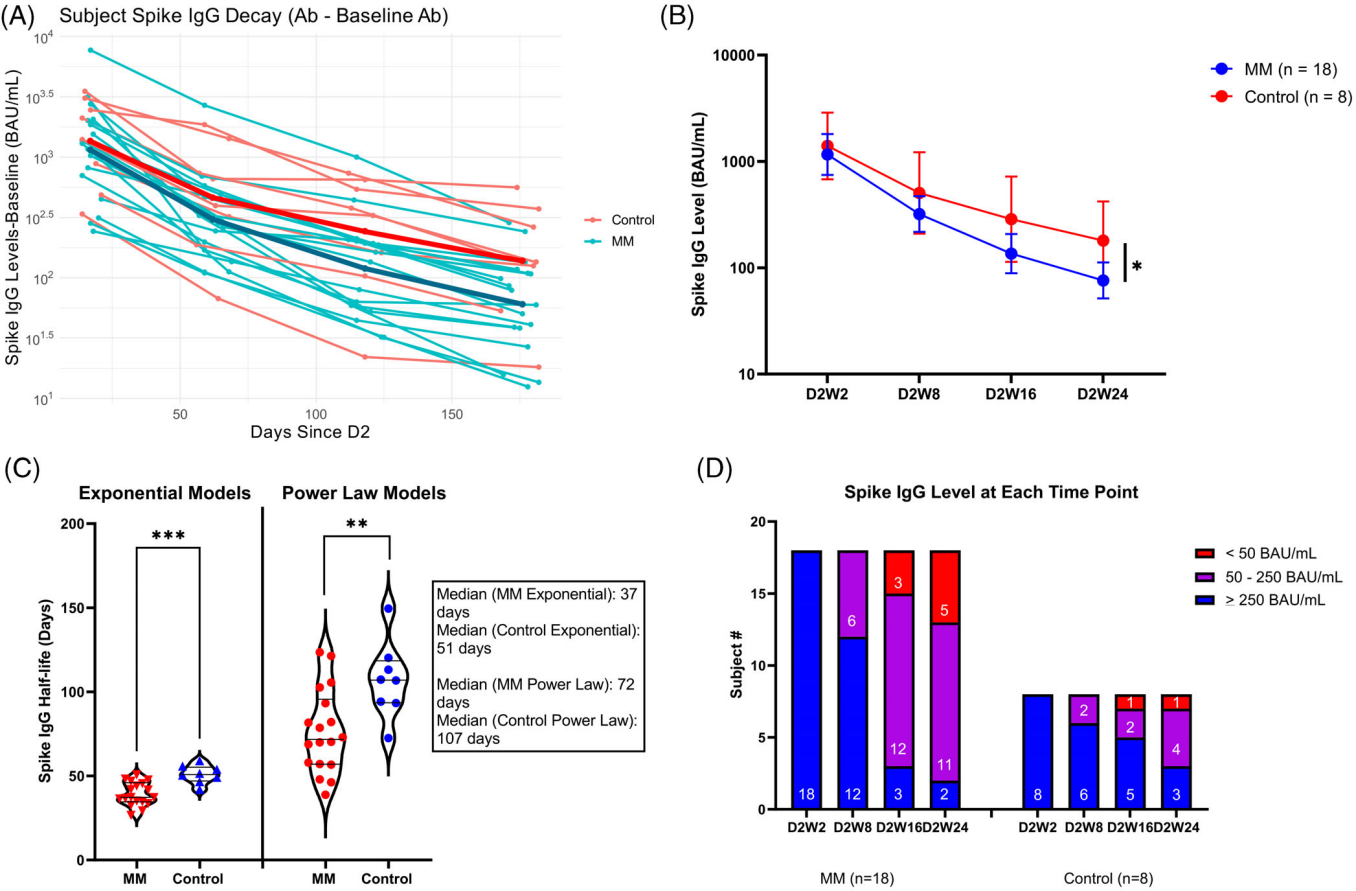
Antibody levels and half‐lives. Antibody levels were determined at serial intervals following dose 2: 2–3 weeks (D2W2), 8–9 weeks (D2W8), 16–17 weeks (D2W16), and 23–24 weeks (D2W24). (A) Raw declining antibody levels following dose 2 in multiple myeloma (MM) patients (blue) and healthy controls (red) with geomean levels from each group in thicker lines. (B) Geomean antibody levels and 95% CI throughout the study. (C) Violin plots of half‐lives calculated by either exponential or power law methods. (D) Spike IgG level groupings throughout the study among patients (left) and controls (right). Antibody levels were log‐transformed prior to any statistical analysis. Comparisons in (B) and (C) done by unpaired *t* tests with significant comparisons indicated by *p < .05; **p < .01; ***p < .001. D2W2, D2W8, and D2W16 comparisons were not significant between groups.

Anti‐spike antibody levels from 2, 8, 16, and 24 weeks post‐second dose vaccination are shown in Figure 2A,B, and Table 1. Patients had lower antibody levels at week 2 (geomean 1161 BAU/mL) as compared to controls (1835 BAU/mL) that were not statistically significant (*p* = .62). The geometric mean antibody levels among patients relative to week 2 declined 3.6‐, 8.6‐, and 15.3‐fold at 8‐, 16‐, and 24‐weeks post vaccination, respectively. In controls, the fold reduction in antibody levels was less (2.8‐, 4.9‐, and 7.77 at 8, 16, and 24 weeks, respectively). Half‐lives were estimated using two methods: the power law method and exponential decay. The former is better suited for modeling antibody decay following vaccination as it accounts for ongoing antibody production by plasma cells and memory B‐cells,^9^ while the latter is best suited for modeling antibody decreases following monoclonal antibody infusions, which lack ongoing antibody production. Using power‐law, median half‐lives were 72 days (IQR [interquartile range] 57–90) among MM patients and longer (median 107 days; IQR 94–115) in controls (*p* = .0070 by unpaired *t* test). Both results are within the range of antibody decay following vaccination among healthy individuals, with a reported range of 62–118 days (based on 95% CI) among patients aged >55 with no difference between ages 56–70 and age >70.^8^ The median exponential decay antibody half‐life in MM patients was 37 days (IQR 35–45), which was significantly less than the median 51‐day (IQR 48–54) half‐life in controls (*p* = .0004; Figure 2C and Table 1). MM patients had more variability in their antibody decay rates compared to healthy controls, with power‐law half‐lives ranging from 39 to 124 days in myeloma patients versus 72–149 days in healthy controls (Tables 1 and S1). Controls were significantly younger than patients by 8 years (Table 1), and the results of an ANCOVA test demonstrated that patients' half‐lives were still statistically significantly lower than controls after controlling for age differences (*p* = .03 for power law and *p* = .003 for exponential decay; Table 1).

**TABLE 1:** Participant information.

	Patients (*n* = 18)	Controls (*n* = 8)	*p* Value
Mean age, years (range)	70.0 (62–82)	62 (53–75)	*p* = .018
# Female (%)	9 (50%)	5 (63%)	*p* = .87
Mean power law half‐life, days (range)	72 (39–124)	107 (72–149)	*p* = .0070
		Age‐controlled:	*p* = .03
Mean exponential half‐life, days (range)	37 (27–51)	51 (42–59)	*p* = .0004
		Age‐controlled:	*p* = .003
D2W2 antibody geomean BAU/mL (range)	1161 (258–7716)	1835 (346–3523)	*p* = .62
D2W8 antibody geomean BAU/mL (range)	320 (113–2705)	758 (76–2002)	*p* = .23
D2W16 antibody geomean BAU/mL (range)	136 (40–1017)	417 (31–828)	*p* = .073
D2W24 antibody geomean BAU/mL (range)	76 (21–305)	261 (27–623)	*p* = .027

*Note*: D2W2 indicates 2 weeks after the second dose; D2W8 indicates 8 weeks, and so forth. Antibody levels indicated as spike‐binding arbitrary units (standardized to WHO 20/136 sera), with geometric means displayed (compared to means, these more accurately reflect the logarithmic concentration and decay of antibodies). Antibody measurements were log‐transformed prior to statistical comparisons. *p* Values indicated on the right by unpaired *t* tests. Given the moderate age‐difference between groups, half‐life comparisons were done both without and with controlling for age (using an analysis of covariance test); both values are displayed for transparency.

Due to shorter half‐lives and reduced peak antibody levels at D2W2, patients with MM showed significantly lower antibody levels at week 24 (Figure 2B and Table 1; *p* = .027), in spite of only including patients with a full response to vaccination. Previously, we had defined a full response as antibody levels above 250 BAU/mL, corresponding to above the 6th percentile of healthy controls and matching the expected initial 94% vaccine efficacy rate in 2020. Only 45% of vaccinated MM patients in our prior study were full responders.^3^ Non‐response was defined as below the assay background of 50 BAU/mL. Partial response (50–250 BAU/mL) yields unclear benefits from vaccination. By D2W24, antibody levels in the majority of both patients and controls had declined to below the full response threshold (Figure 2D). The difference between patients and controls was most pronounced at week 16, at which point 15/18 (83%) of MM patients had dropped into partial or non‐response thresholds as compared to only 3/8 (38%) of controls (*p* = .06 by *Z* test). At D2W24, 5/18 (28%) of MM patients had levels <50 BAU/mL, compared to 1/8 (13%) of controls (*p* = .73).

Only 5/18 (28%) patients had power law half‐lives exceeding the bottom 25% of controls. Interestingly, all 5 had control of their MM, with 4/5 having undetectable monoclonal (M)‐protein throughout the study and the other with a maximum M‐protein of only 0.81 g/dL (Table S2). In contrast, just 4/13 of the remaining patients had undetectable M‐protein, with 6/13 exceeding 0.81 g/dL. This difference was statistically significant by Mann–Whitney *U* test (*p* = .036). The geomean IgG of these 5 patients was 728 BAU/mL at D2W2, which was less than the other patients (1390 BAU/mL; *p* = .17) and lower than controls by unpaired *t* test (1835 BAU/mL; *p* = .20). Despite peak antibody levels that were less than half of the other patients, these 5 patients demonstrated higher geomean antibody levels at the end of the study (D2W24) of 118 BAU/mL, compared to 64 BAU/mL among the remaining patients (*p* = .15). These differences were not statistically significant but may merit further evaluation in a larger cohort. There was no difference in the average age of longer half‐life patients compared to the rest of the patients.

## 4 DISCUSSION

The data from this study demonstrate that the subset of MM patients who respond to mRNA vaccination for SARS‐CoV‐2 have more rapid antibody decay as compared to that of the controls. Nearly all MM patients had antibody levels decline to <250 BAU/mL, a level that is below fully protective, by 24 weeks. In 2020 and early 2021, >147 anti‐spike BAU/mL was associated with protection from infection in healthy individuals^10^ and 126 BAU/mL in a MM patient was associated with incomplete protection from severe disease.^7^ Given their reduced antibody responses to vaccination and shorter half‐lives, most MM patients would be expected to be susceptible to wild‐type SARS‐CoV‐2 infection 4–6 months after vaccination. We noted that patients with the longest half‐lives were more likely to have undetectable M‐protein, but overall observed variable responses to COVID‐19 vaccination with respect to both antibody levels and half‐lives.

The study was limited by lack of blinding. Patients were aware of their anti‐spike IgG levels, prompting some individuals to obtain additional vaccine doses prior to the end and thus were excluded from the study. Nine patients left the study between D2W16 and D2W24. They had lower geomean antibody levels from both D2W2 (783 vs. 1161 BAU/mL; *p* = .27) and D2W16 (88 vs. 136 BAU/mL; *p* = .30) that were not statistically significant at a 0.05 cutoff, so perhaps their antibody levels may have influenced a decision to leave the study in order to have additional vaccination(s), but this did not impact the median half‐life, which was nearly identical in both groups (Table S3). Measuring other immunologic parameters was outside the scope of this work. In other studies, MM patients who developed adequate antibody responses to vaccination for COVID‐19 also developed spike‐specific T‐cells at similar rates to healthy controls.^2^ These T‐cells have longer half‐lives than anti‐spike antibodies and may confer additional protective benefits independent of antibodies.^11^, ^12^

The study was also limited by small sample size. To be able to accurately measure vaccine‐induced antibody half‐lives, antibodies must increase after vaccination and then be allowed subsequently to decay without repeat exposure to the vaccinating antigen (which would boost antibody levels). This study took place during a 6‐month period that included widespread transmission of Beta and Delta SARS‐CoV‐2 variants, resulting in many participants being exposed (and having spike antibodies rise at points after D2W2) as well as causing many to leave the study to receive a booster vaccination. This left us with just 18 patients and 8 controls who had no evidence of interval SARS‐CoV‐2 exposure. Nonetheless, the difference in the patient versus control half‐lives is very unlikely to occur by chance alone, even controlling for the modest age difference in the groups (*p* = .003 for exponential half‐life). A larger sample size would likely provide even greater statistical power to see the difference, as well as to elucidate additional MM‐specific features contributing to decreased half‐lives, but repeating this trial is impossible due to widespread vaccination and prior COVID‐19.The BA.2 strain is 8.4‐ to 27‐fold less susceptible to antibody neutralization compared to wild‐type SARS‐CoV‐2. Therefore, much higher antibody levels may be necessary to prevent infection and hospitalization from infection with this strain.^13^ Booster doses were effective at reducing healthcare encounters and hospitalization due to the BA.1 strain in healthy individuals, but efficacy dropped rapidly from 2–3 months (81%) to 4 months (66%) post‐vaccination (14). Our data suggests that MM patients will be unable to maintain high titer antibody levels needed to prevent infection and hospitalization from BA.2 and should continue to socially distance after receiving boosters. Due to a rapid reduction in vaccine‐induced SARS‐CoV‐2 antibodies in MM patients, those with active disease should be considered for prophylactic monoclonal antibodies regardless of vaccine‐induced anti‐spike antibody levels. Tixagevimab/cilgavimab is one such antibody combination that had been used effectively until the emergence of the BQ.1.1 and later variants, so unfortunately we await the development of future monoclonal antibody prophylactics with efficacy against current strains.^14^, ^15^ If vaccines are developed with better efficacy against circulating SARS‐CoV‐2 variants, our results suggest that myeloma patients are likely to require more frequent vaccination to compensate for often having nonprotective antibody levels and shorter half‐lives.

## 5 CONCLUSION

Some patients with multiple myeloma have similar antibody responses to mRNA vaccination for COVID‐19 as healthy individuals; however, we found that their vaccine‐induced antibodies are shorter lived. With ongoing circulation of omicron‐family SARS‐CoV‐2 variants, MM patients will likely remain susceptible to infection and should continue to rely on social distancing to prevent COVID‐19. As new mRNA vaccines are developed for different diseases, dedicated studies on MM patients are critical to ensure that they have adequate antibody responses to vaccination, with special attention to their waning antibody levels over a 6‐month follow‐up period.

## AUTHOR CONTRIBUTIONS

**Samuel David Stampfer:** Conceptualization (equal); formal analysis (equal); investigation (supporting); methodology (lead); supervision (equal); validation (lead); visualization (lead); writing – original draft (lead); writing – review and editing (lead). **Sean Bujarski:** Data curation (lead); formal analysis (equal); investigation (equal); validation (supporting). **Marissa‐Skye Goldwater:** Data curation (supporting); investigation (lead); methodology (supporting); validation (supporting). **Scott Jew:** Formal analysis (equal). **Bernard Sean Regidor:** Data curation (equal). **Haiming Chen:** Data curation (equal); investigation (supporting); methodology (supporting). **Ning Xu:** Data curation (equal); investigation (supporting); methodology (supporting). **Mingjie Li:** Data curation (equal); investigation (supporting); methodology (supporting). **Eddie Fung:** Data curation (supporting). **Regina A. Swift:** Data curation (equal). **Bethany Beatty:** Data curation (equal). **Shahrooz Eshaghian:** Data curation (equal). **James R. Berenson:** Conceptualization (equal); supervision (equal); visualization (equal); writing – original draft (supporting); writing – review and editing (supporting).

## CONFLICT OF INTEREST STATEMENT

The authors have stated explicitly that there are no conflicts of interest in connection with this article.

## ETHICS STATEMENT

All procedures met the Helsinki Declaration of the World Medical Association ethical standards. Written consent was obtained from participants.

## Supporting information


**TABLE S1.** Analyzed data from patients and controls. Raw source data is included separately in Source Data

**TABLE S2.** Comparison of MM patients with normal vs shortened half‐lives. The top 5 patients' half‐lives were comparable to controls and analyzed separately from the other 13 patients. M‐protein levels were taken at all points in the study and individual patient averages were compared. Antibody levels were log‐transformed prior to statistical tests. *p*‐values are indicated on the right by unpaired *t* tests, except for M‐protein where a Mann‐Whitney test was used given data that is not normally distributed.

**TABLE S3.** Comparison of MM patients at D2W16. 18 patients remained in the study through D2W24 while 9 patients were lost to follow‐up after week 16 (either through receiving booster vaccination, contracting COVID‐19, or missing their week 24 blood draw). Half‐lives listed are through week 16, allowing for exponential half‐life calculation only. One patient in the loss‐to‐followup group appeared to be an outlier with a very prolonged half‐life of 151 days (significantly affecting the mean), but perhaps this would have normalized by week 24. For transparency and clarity, both mean and median values are displayed for each group. *p*‐values are indicated on the right by unpaired *t* tests, with antibody‐values log‐transformed prior to statistical analysis.

## Data Availability

All data generated or analyzed during this study are included in this published article and its supplementary information files.
