# Supplementary material for: Loss of anti‐spike antibodies following mRNA vaccination for COVID‐19 among patients with multiple myeloma
Source: Cancer Rep (Hoboken). 2023 Mar 9;6(5):e1803. doi: 10.1002/cnr2.1803 (PMC10172160; doi:10.1002/cnr2.1803)
Supplement: Supplementary file 2 — TABLE S2. Comparison of MM patients with normal vs shortened half‐lives. The top 5 patients' half‐lives were comparable to controls and analyzed separately from the other 13 patients. M‐protein levels were taken at all points in the study and individual patient averages were compared. Antibody levels were log‐transformed prior to statistical tests. p‐values are indicated on the right by unpaired t tests, except for M‐protein where a Mann‐Whitney test was used given data that is not normally distributed. [file CNR2-6-e1803-s002.pdf]

	Normal half-lives (N=5)	Shortened half-lives (N=13)	p-value
Mean Age, years (range)	71.0 (61 - 80)	70.0 (61 - 84)	p = 0.87
Mean Power law half-life, days (range)	106 (93 - 124)	69 (39 – 82)	p < 0.0001
Mean exponential half-life, days (range)	48 (46 - 51)	36 (27 - 44)	p < 0.0001
Average M-protein, g/dL (range)	0.13 (0 - 0.81)	1.27 (0 - 4.2)	p = 0.036
D2W2 antibody geomean BAU/mL (range)	728 (258 - 2053)	1320 (286 - 7716)	p = 0.17
D2W8 antibody geomean BAU/mL (range)	338 (152 - 733)	314 (113 - 2705)	p = 0.85
D2W16 antibody geomean BAU/mL (range)	206 (95 - 478)	116 (40 - 1017)	p = 0.21
D2W24 antibody geomean BAU/mL (range)	118 (56 - 279)	64 (21 - 305)	p = 0.15

Supplemental Table 2 Comparison of MM patients with normal vs shortened half-lives. The top 5 patients' half-lives were comparable to controls and analyzed separately from the other 13 patients. M-protein levels were taken at all points in the study and individual patient averages were compared. Antibody levels were log-transformed prior to statistical tests. p-values are indicated on the right by unpaired t-tests, except for M-protein where a Mann-Whitney test was used given data that is not normally distributed.
