# Supplementary material for: Loss of anti‐spike antibodies following mRNA vaccination for COVID‐19 among patients with multiple myeloma
Source: Cancer Rep (Hoboken). 2023 Mar 9;6(5):e1803. doi: 10.1002/cnr2.1803 (PMC10172160; doi:10.1002/cnr2.1803)
Supplement: Supplementary file 3 — TABLE S3. Comparison of MM patients at D2W16. 18 patients remained in the study through D2W24 while 9 patients were lost to follow‐up after week 16 (either through receiving booster vaccination, contracting COVID‐19, or missing their week 24 blood draw). Half‐lives listed are through week 16, allowing for exponential half‐life calculation only. One patient in the loss‐to‐followup group appeared to be an outlier with a very prolonged half‐life of 151 days (significantly affecting the mean), but perhaps this would have normalized by week 24. For transparency and clarity, both mean and median values are displayed for each group. p‐values are indicated on the right by unpaired t tests, with antibody‐values log‐transformed prior to statistical analysis. [file CNR2-6-e1803-s001.pdf]

	Not lost to followup (N = 18)	Lost to followup (N = 9)	p-value
Median exponential half-life, days (range)	32.4 (19 – 90)	33.4 (16 – 151)	p = 0.52
Mean exponential half life, days	37.1	44.4	
D2W2 antibody geomean BAU/mL (range)	1161 (258 - 7716)	783 (262 - 2842)	p = 0.27
D2W8 antibody geomean BAU/mL (range)	320 (113 - 2705)	200 (12 – 800)	p = 0.26
D2W16 antibody geomean BAU/mL (range)	136 (40 - 1017)	88 (9 - 304)	p = 0.30

Supplemental Table 3 Comparison of MM patients at D2W16. 18 patients remained in the study through D2W24 while 9 patients were lost to follow-up after week 16 (either through receiving booster vaccination, contracting COVID-19, or missing their week 24 blood draw). Half-lives listed are through week 16, allowing for exponential half-life calculation only. One patient in the loss-to-followup group appeared to be an outlier with a very prolonged half-life of 151 days (significantly affecting the mean), but perhaps this would have normalized by week 24. For transparency and clarity, both mean and median values are displayed for each group. p-values are indicated on the right by unpaired t-tests, with antibody-values log-transformed prior to statistical analysis.
